# Clinical findings in Brazilian patients with adult GM1 gangliosidosis

**DOI:** 10.1002/jmd2.12067

**Published:** 2019-07-17

**Authors:** Luciana Giugliani, Carlos Eduardo Steiner, Chong Ae Kim, Charles Marques Lourenço, Mara Lucia Schmitz Ferreira Santos, Carolina Fischinger Moura de Souza, Ana Carolina Brusius‐Facchin, Guilherme Baldo, Mariluce Riegel, Roberto Giugliani

**Affiliations:** ^1^ National Institute of Population Medical Genetics (INAGEMP) Porto Alegre Brazil; ^2^ Department of Medical Genetics Faculdade de Medicina, UNICAMP Campinas Brazil; ^3^ Instituto da Criança Hospital das Clínicas, FM, USP São Paulo Brazil; ^4^ Centro Universitário Estácio de Ribeirão Preto Ribeirão Preto Brazil; ^5^ Hospital Infantil Pequeno Príncipe Curitiba Brazil; ^6^ Medical Genetics Service, HCPA Porto Alegre Brazil; ^7^ Gene Therapy Center, HCPA Porto Alegre Brazil; ^8^ Department of Physiology UFRGS Porto Alegre Brazil; ^9^ Post‐Graduate Program in Physiology UFRGS Porto Alegre Brazil; ^10^ Post‐Graduate Program in Genetics and Molecular Biology UFRGS Porto Alegre Brazil; ^11^ Department of Genetics UFRGS Porto Alegre Brazil

**Keywords:** beta‐galactosidase deficiency, Brazil, GM1 gangliosidosis, INAGEMP, late onset, sphingolipidosis

## Abstract

GM1 gangliosidosis is a lysosomal storage disorder caused by β‐galactosidase deficiency. To date, prospective studies for GM1 gangliosidosis are not available, and only a few have focused on the adult form. This retrospective cross‐sectional study focused on clinical findings in Brazilian patients with the adult form of GM1 gangliosidosis collected over 2 years. Ten subjects were included in the study. Eight were males and two females, with median age at diagnosis of 11.5 years (IQR, 4‐34 years). Short stature and weight below normal were seen in five out of the six patients with data available. Radiological findings revealed that the most frequent skeletal abnormalities were beaked vertebrae, followed by hip dysplasia, and platyspondyly. Neurological examination revealed that dystonia and swallowing problems were the most frequently reported. None of the patients presented hyperkinesia, truncal hypertonia, Parkinsonism, or spinal cord compression. Clinical evaluation revealed impairment in activities of cognitive/intellectual development and behavioral/psychiatric disorders in all nine subjects with data available. Language/speech impairment (dysarthria) was found in 8/9 patients, fine motor and gross motor impairments were reported in 7/9 and 5/9 patients, respectively. Impairment of cognition and daily life activities were seen in 7/9 individuals. Our findings failed to clearly identify typical early or late alterations presented in GM1 gangliosidosis patients, which confirms that it is a very heterogeneous condition with wide phenotypic variability. This should be taken into account in the evaluation of future therapies for this challenging condition.

## 1 INTRODUCTION

Gangliosidoses are autosomal recessive inherited metabolic diseases in which accumulation of gangliosides (glycosphingolipids containing one or more sialic acid residues) in the central nervous system (CNS) leads to severe and progressive neurological impairment.^1^, ^2^ GM1 gangliosidosis is a lysosomal storage disorder caused by β‐galactosidase deficiency. Accumulation of GM1 ganglioside and related glycoconjugates in the lysosomes leads to lysosomal swelling, and cellular and organ.^1^, ^2^

Primary β‐galactosidase deficiency is also found in patients with Morquio disease type B (mucopolysaccharidosis IVB; MPS IVB). In this condition, the genetic mutation affects the catalytic activity related to the substrate keratan sulfate, causing a disease marked by bone dysplasia usually without neurological involvement.^3^ Patients with intermediate phenotypes (clinical findings of MPS IVB and GM1 gangliosidosis) have been reported.^4^ Secondary β‐galactosidase deficiency may occur in galactosialidosis (deficiency of the PPCA protein, which protects β‐galactosidase and neuraminidase from early degradation) and mucolipidoses II and III (deficiency of *N*‐acetyl‐glucosamine‐phosphate transferase, which is required for the normal trafficking of β‐galactosidase and other lysosomal enzymes to the lysosome) resulting in increased activity in plasma and decreased activity in tissues.^2^

GM1 gangliosidosis is a neurodegenerative condition classified as type I (infantile, OMIM #230500), type II (late infantile/juvenile, OMIM # 23060), and type III (adult, OMIM # 23650) clinical forms.^5^ The severe infantile phenotype (type I) is characterized by psychomotor manifestations by the age of 6 months, visceromegaly, facial and skeletal abnormalities, severe progressive CNS degeneration, and death by 1 to 2 years of age.^5^ The abnormalities in type II usually start between 7 months and 3 years of age with slowly progressing neurological signs, including early gait abnormalities, strabismus, muscle weakness, seizures, lethargy, and recurrent bronchopneumonia.^5^ Skeletal changes are less severe than observed in type I.

The adult form or chronic late‐onset variant (type III) has a less severe phenotype, with onset usually between 3 and 30 years, although cases with earlier onset and protracted course may occur. It is characterized by progressive neurological impairment, with cerebellar dysfunction, dystonia, slurred speech, and mild vertebral deformities.^2^, ^5^ The estimated incidence of GM1 gangliosidosis is reported as 1:100 000 to 200 000 live births.^6^ However, the early infantile form seems to be much more frequent in Brazil,^7^ with an incidence of 1:13 317 live births, and a carrier frequency of 1:58.^8^

The GLB1 protein is encoded by the *GLB1* gene (E.C.3.2.1.23; MIM 230500), mapping to chromosome 3p21.33. According to the Human Gene Mutation Database (HGMD), more than 160 mutations have been described as causative of GM1 gangliosidosis,^6^ not including mutations related to Morquio B disease.

To date, natural history studies of GM1 gangliosidosis have been based on retrospective data collected through surveys.^9^ Prospective studies for GM1 gangliosidosis are not available. However, a few case series have been published describing several findings in these patients.^6^, ^10^, ^11^

Currently, only symptomatic and supportive treatments are available for GM1 gangliosidosis.^10^ Different strategies have been explored to treat this disease, and they include hematopoietic stem cell transplantation (HSCT), substrate reduction therapy, and gene therapy. *N*‐butyldeoxynojirimycin (NB‐DNJ, miglustat)‐mediated substrate reduction therapy has been approved to treat patients with type 1 Gaucher disease and Niemann‐Pick disease type C and has been used experimentally in late‐onset Sandhoff disease.^12^, ^13^, ^14^, ^15^ Interesting results have also been reported in a mouse model of GM1 gangliosidosis.^16^ Research is underway in animal models to evaluate gene and enzyme replacement therapies for the treatment of this disease.^6^, ^9^, ^11^, ^17^ However, robust data about the natural history of GM1 patients are necessary to evaluate treatment efficacy. This study aimed to contribute to the better understanding of the adult form of GM1 gangliosidosis, presenting a retrospective characterization of a sample of Brazilian patients by focusing on clinical features.

## 2 MATERIALS AND METHODS

### 2.1 Patients and ethics

The study was approved by the Ethics Research Committee of Hospital de Clínicas de Porto Alegre (HCPA), followed the Declaration of Helsinki, and the standards established by the authors' Institutional Review Board and granting agency. This is a retrospective cross‐sectional study involving a series of cases. Data collection occurred from 2015 to 2017.

Subjects were recruited at the Inborn Errors of Metabolism outpatient clinics at HCPA and at services associated with the LSD Brazil Network. Patients were referred from different regions in Brazil with a biochemical and/or genetic diagnosis of GM1 gangliosidosis and with clinical manifestations compatible with the adult form. Patients with confirmed diagnosis, of both genders, any age, and any ethnicity were enrolled.

### 2.2 Clinical features

Clinical, phenotypic, and biochemical data were obtained from patient records and recorded in a specific form.

### 2.3 Molecular genetics analysis

Molecular genetics data were obtained from patient records and consisted of most of the mutations already published.^8^, ^18^, ^19^, ^20^, ^21^, ^22^

### 2.4 Statistical analysis

Statistical analyses were conducted using the REDCap software, which is specifically designed for medical data collection. REDCap is web‐based, uses 128‐bit data encryption, and provides role‐based security by requiring a user ID and password for access. All subject data were automatically identified by a computer‐generated code to which only the researchers involved in the survey had access. In this data analysis, we describe the number of GM1‐diagnosed cases and the frequency and type of symptoms that occurred according to the age of the patients. We also describe the clinical and biochemical phenotypes and patient genotypes. Categorical variables are expressed as absolute and relative frequencies and continuous variables as mean ± SD or median (interquartile range). The chi‐square test was used to test for associations between categorical variables. Other nonparametric tests were employed as needed to evaluate potential associations among study variables. Results were considered significant at *P* < .05.

## 3 RESULTS

This is a retrospective cross‐sectional study in which clinical information was obtained from patients' records. Ten patients were included, eight males and two females. All these patients had the adult form of the disease. The median age at diagnosis was 11.5 years (IQR, 4‐34 years). Consanguinity was reported in 2/10 patients. The age at onset of symptoms ranged from 0.5 to 7 years (mean = 3.55 years). The sample profile is described in Table 1.

**Table 1:** Demographic features in a Brazilian sample of GM1 gangliosidosis (n = 10)

Patient ID	Gender^a^	Parents consanguinity	Educational background	Amino acid change^b^	cDNA change^c^	First symptoms observed	Age at first symptoms (years)	Age at clinical evaluation (years)	Biochemical
PA 001	M	No	Elementary school	NA	NA	Speech delay at 4 years old. Gait abnormalities (reported as “duck walk”).	4	10	Normal uGAGs with abnormal uOLS and deficient activity of beta‐galactosidase in leucocytes
PA 002^d^	M	No	Special aid school	p.Arg59His/p.Arg201His	c.176G>A/c.602G>A	Sudden change in behavior with hyperactivity and aggressiveness. In addition, progressive dyslalia, motor incoordination, and gait pattern alteration.	7	27	Normal uGAGs with keratan sulfate identified in urine; normal uOLS and deficient activity of beta‐galactosidase in leucocytes
PA 007	M	No	Elementary school	NA	NA	Learning difficulties at school as well as some fine motor skill and speech impairments. Agitation during sleep periods and difficulties swallowing with occasional gagging.	4	10	No information about uGAGs, abnormal uOLS and and deficient activity of beta‐galactosidase in leucocytes
PA 008^d^	M	No	Special aid school	p.Arg59His/ p.Trp527Leufs*5	c.176G>A/c.1577dupG	Neurodevelopmental delay relative to the dizygotic twin. At 30 months: gait instability and progressive skeletal deformity.	2	34	Normal uGAGs with abnormal uOLS and deficient activity of beta‐galactosidase in leucocytes
PA 009	M	No	Elementary school	p.Thr500Ala/ p.Trp527Leufs*5	c.1498A>G/c.1577dupG	Progressive speech impairment, gait abnormality (lateral displacement and wide base) at 4 years and mild paraparesis at 5 years old.	3	34	No information about uGAGs or uOLS, with deficient activity of beta‐galactosidase in leucocytes
PA 010	F	No	Special aid school	p.Ile284Asnfs*12/p.Tyr64Cys	c.848dupC/c.191A>G	Gain in plantar flexion, scoliosis, and often falls.	2	19	No information about uGAGs or uOLS, with deficient activity of beta‐galactosidase in leucocytes
PA 012	M	No	NA	NA	NA	Fever and convulsive crises.	5	13	No information about uGAGs, abnormal uOLS and and deficient activity of beta‐galactosidase in leucocytes
PA 015	M	No	Special aid school	NA	NA	Gait delay and recurrent airway infections.	2	5	Normal uGAGs, no information on uOLS and and deficient activity of beta‐galactosidase in leucocytes
PA 016	M	Yes	Special aid school	NA	NA	Gibbous and progressive loss of walking ability.	3	10	No information about uGAGs or uOLS, with deficient activity of beta‐galactosidase in leucocytes
PA 017	F	Yes	NA	NA	NA	Regression of neurodevelopment at 5 months and hepatomegaly.	0.5	4	No information about uGAGs or uOLS, with deficient activity of beta‐galactosidase in leucocytes

Abbreviation: NA, not analyzed/not available.

a Gender: F: female; M: male.

b Amino acid change according to protein sequence NP_000395.2.

c cDNA numbering +1 corresponds to the “A” of the first ATG translation initiation codon with RefSeq NM_000404.2.

d Patient included in the report of Reference 18.

Regarding molecular analysis, out of the 10 patients included in the study, we only had access to genotype data for four. All of these mutations have been previously reported.^6^, ^8^, ^10^, ^19^, ^21^ The mutations identified in the GLB1 gene are shown in Table 1.

Short stature and low weight were observed in 5/6 patients with data available. Increased liver size was reported in two patients. Cardiomyopathy (nonspecific), valvular insufficiency, sinusitis, coarse voice, corneal clouding, and hearing impairment, were seen in one patient. Other signs previously described in lysosomal storage diseases such as skeletal findings, coarse faces, large head and genu varum, were also rare. Short neck, protrusion of sternum, barrel‐shaped thorax, joint stiffness, joint contractures, waddling gait, genu valgum, and elbow or shoulder problems were seen in two individuals. Scoliosis or gibbus and kyphosis were present in 7/10 and 4/10 patients with data available, respectively. Radiological findings revealed that the most frequent skeletal abnormalities were beaked vertebrae (5/9), followed by hip dysplasia (4/9), platyspondyly (4/9), osteopenia (2/9), J‐shaped sella turcica (2/9), and odontoid hypoplasia (1/9; Table 2).

**Table 2:** Clinical features in Brazilian patients with adult GM1 gangliosidosis (n = 10), from youngest (PA 017) to oldest (PA008)

Patient	PA 017	PA 015	PA 016	PA 007	PA 001	PA 012	PA 010	PA 002	PA 009	PA 008	Total
Short stature	NA	NA	NA	+	−	NA	+	+	+	+	5/6
Low weight	NA	NA	NA	+	−	NA	+	+	+	+	5/6
Hepatomegaly	+	+	−	−	NA	−	−	−	−	−	2/9
Splenomegaly	−	−	−	−	+	−	−	−	−	−	1/10
Heart X‐ray	−	−	−	+	NA	+	NA	NA	NA	−	2/6
Heart ultrasound											
Cardiomyopathy (nonspecific)	−	−	+	−	−	−	−	−	−	−	1/9
Valvular insufficiency	−	−	−	−	−	+	−	−	−	−	1/9
Ear nose throat airways											
Adenoid hyperplasia	NA	+	+	−	+	+	−	−	−	−	4/9
Tonsillar hyperplasia	NA	+	+	−	+	+	−	−	−	−	4/9
Sinusitis	NA	−	−	−	+	−	−	−	−	−	1/9
Hoarse voice	NA	−	−	−	+	−	−	−	−	−	1/9
Pulmonary X‐ray											
Atelectasis	−	−	+	−	−	−	NA	−	−	NA	1/8
Interstitial markings	−	+	−	−	−	+	NA	−	−	NA	2/8
Laryngo‐tracheoscopy											
Tracheal collapse	−	NA	+	NA	NA	NA	−	NA	−	−	1/5
Skeletal findings clinical											
Coarse face	+	−	−	−	−	−	−	−	−	−	1/10
Large head	−	−	−	−	−	+	−	−	−	−	1/10
Short neck	−	+	−	−	−	+	−	−	−	−	2/10
Protrusion of sternum	−	−	−	−	−	−	−	+	−	+	2/10
Barrel‐shaped thorax	−	−	−	−	−	−	−	+	−	+	2/10
Kyphosis	−	−	−	−	+	−	−	+	+	+	4/10
Scoliosis or gibbous	−	−	+	+	+	+	+	+	−	+	7/10
Joint stiffness	−	−	−	−	−	+	+	−	−	−	2/10
Joint contractures	−	−	−	−	−	−	+	−	+	−	2/10
Hyperextensive joints	−	−	+	+	+	−	−	−	−	−	3/10
Waddling gait	−	−	−	−	−	−	−	+	−	+	2/10
Genu valgum	−	−	−	−	+	−	+	−	−	−	2/10
Genu varum	−	−	+	−	−	−	−	−	−	−	1/10
Elbow or shoulder problems	−	−	−	−	−	−	−	+	−	+	2/10
Skeletal findings radiological											
J‐shaped sella	−	−	−	−	−	−	NA	+	−	+	2/9
Odontoid hypoplasia	−	−	−	−	−	−	NA	−	−	+	1/9
Platyspondyly	−	+	+	−	−	−	NA	−	+	+	4/9
Beaked vertebrae	−	+	+	−	−	+	NA	+	−	+	5/9
Hip dysplasia	−	−	+	−	−	−	NA	+	+	+	4/9
Osteopenia	−	−	+	−	−	+	NA		−	−	2/9
Surgical procedure											
Hip	−	−	−	−	−	−	−	+	+	−	2/10
Knee	−	−	−	−	−	−	−	−	+	−	1/10
Spine	−	−	−	−	−	−	−	+	−	+	2/10
Tonsillectomy	−	−	−	−	+	+	−	−	−	−	2/10
Ear tubes	−	+	+	−	−	−	−	−	−	−	2/10
Teeth/palate/gum											
Broad, flat hard palate	−	−	−	−	−	+	−	−	−	+	2/10
Pain											
Hip	NA	NA	−	−	−	−	−	+	−	+	2/8
Long bones	NA	NA	−	−	−	−	−	−	+	−	1/8
Ability to walk											
Requires walking aid	NA	−	+	+	+	+	+	+	+	+	8/9
Requires wheelchair	NA	−	−	−	−	+	+	+	+	+	5/9
Bedridden	NA	−	−	−	−	−	−	−	−	+	1/9
Sleep problems											
Obstructive apnea	NA	+	+	−	−	−	−	NA	−	−	2/8
Behavioral insomnia	NA	−	−	−	−	+	−	+	+	+	3/8
Eye abnormalities											
Corneal clouding	−	−	+	NA	−	−	−	−	−	−	1/9
Hearing impairment											
Conductive hearing impairment	NA	−	+	−	−	−	−	−	NA	NA	1/7

Abbreviations: +, present; −, absent; NA, not available.

*Note*: Other aspects evaluated but not found (absent in all patients) were as follows: Heart ultrasound: hypertrophic cardiomyopathy, dilated cardiomyopathy, valvular stenosis, coronary abnormalities, pericardial effusion; ear‐nose‐throat: macroglossia; sero‐/mucotympanon, supra/infraglottic narrowing, tracheomalacia habitual neck extension (to increase airway patency); Pulmonary X‐ray: air trap, central infiltration; Pulmonary function: obstructive, restrictive, obstructive/restrictive; Teeth/palate/gum: gingival hyperplasia; Spinal cord compression (radiological): thoracic, lumbar; Eye abnormalities: cataract, Cherry red spot, optic atrophy, retinopathy.

Clinical evaluation revealed that the majority of patients needed help to walk: eight and five patients out of nine with data available required a walking aid and wheelchair, respectively (Table 2).

Neurological examination revealed dystonia (6/10) and swallowing problems (6/10) as the most frequent symptoms, followed by hyperreflexia (5/10), truncal hypotonia (5/10), spasticity (4/10), ataxia (4/10), and distal hypotonia (3/10). Other findings occurred in only one or two individuals. No patient presented hyperkinesia, truncal hypertonia, Parkinsonism, or spinal cord compression (Table 3).

**Table 3:** Neurological findings in Brazilian patients with adult GM1 gangliosidosis patients (n = 10)

Patient	PA 017	PA 015	PA 016	PA 007	PA 001	PA 012	PA 010	PA 002	PA 009	PA 008	Total
Neurologic exam											
Spasticity	−	−	−	−	+	+	−	−	+	+	4/10
Dystonia	−	+	+	−	−	−	+	+	+	+	6/10
Athetosis	−	−	+	−	−	−	+	−	−	−	2/10
Chorea	−	−	−	−	−	−	+	−	−	−	1/10
Ataxia	−	+	−	+	−	−	+	+	−	−	4/10
Hypokinesis	+	−	−	−	−	−	−	−	−	−	1/10
Tremor	−	−	−	−	−	−	−	−	−	+	1/10
Truncal hypotonia	+	+	+	−	+	−	+	−	−	−	5/10
Distal hypotonia	+	−	+	+	−	−	−	−	−	−	3/10
Distal hypertonia	−	−	−	−	+	−	−	−	−	−	1/10
Hyperreflexia	−	+	+	−	+	−	−	−	+	+	5/10
Clonus	−	−	−	−	−	−	−	−	−	+	1/10
Hypo/areflexia	+	−	−	−	−	−	+	−	−	−	2/10
Positive plantar sign	−	−	−	−	+	−	−	−	−	−	1/10
Nystagmus	−	−	+	−	−	−	−	−	−	−	1/10
Swallowing problems	+	−	+	−	+	−	+	−	+	+	6/10
Muscular atrophy	−	−	−	−	−	−	+	−	−	+	2/10
Brain Neuroimaging (CT/MRI)											
General brain atrophy	+	−	−	−	+	−	NA	−	−	−	2/9
Subcortical white matter changes	−	−	−	−	+	+	NA	−	−	−	2/9
Ventriculomegaly	−	−	−	−	−	−	NA	−		+	1/9
Basal ganglia: abnormal signal intensity	−	−	−	−	+	−	NA	−	+	+	3/9
Basal ganglia changes: atrophy	−	−	−	−	+	−	NA	−	+	+	3/9
Hypomyelination	+	−	−	−	−	−	NA	−	−	−	1/9
Neuroimaging spinal											
Spinal cord atrophy	NA	NA	NA	NA	NA	NA	NA	−	NA	+	½
Spinal compression clinical	NA	−	−	+	−				+	+	
Lower limb weakness		−	−	+	−	NA	NA	NA	−	−	1/6
Upper limb weakness		−	−	+	−	NA	NA	NA	−	−	1/6
Lower limb spasticity		−	−	−	−	NA	NA	NA	+	+	2/6
Upper limb spasticity		−	−	−	−	NA	NA	NA	−	+	1/6
Spinal cord compression radiological											
Cervical	NA	−	−	−	−	NA	NA	NA	NA	+	1/5
Cognitive/Intellectual Development											
Gross motor	+	−	+	−	+	−	+	−	NA	+	5/9
Fine motor	+	+	+	−	+	−	+	+	NA	+	7/9
Language/speech disturbance (dysathria)	+	+	+	+	+	−	+	+	NA	+	8/9
Social interaction	−	+	+	−	−	−	+	+	NA	+	5/9
Cognitive delay	−	+	+	+	+	−	+	+	NA	+	7/9
Self‐supporting behavior (eating, toileting)	−	−	−	+	+	−	+	+	NA	+	5/9
Activities of daily life	−	+	+	+	+	−	+	+	NA	+	7/9
Emotions/behavior	−		−	−	+	−	−	−	NA	+	2/9
Behavioral disorders/Psychiatric disorders											
ADHD	NA	−	−	−	+	−	−	+	−	−	2/9
Learning disability	NA	−	−	−	+	−	−	+	−	−	2/9
Autism spectrum	NA	−	−	−	+	−	−	+	−	−	2/9
Depression	NA	−	−	−	−	−	+	−	−	−	1/9
Anxiety	NA	−	−	−	+	−	+	−	+	−	3/9
Aggressive behavior	NA	−	−	−	−	−	+	+	−	−	2/9
Obsessive compulsive disorder (OCD)	NA	−	−	−	−	−	−	−	+	−	1/9
Epilepsy											
Myoclonic	−	+	−	−	−	−	−	−	−	−	1/10
Infantile spasms	−	−	+	−	−	+	−	−	−	−	2/10
Tonic clonic	+	−	−	−	−	−	−	−	−	−	1/10

*Note*: Others aspects evaluated but not found (absent in all patients) were as follows: Neurologic exam: hyperkinesis, truncal hypertonia; Neuroimaging spinal: spinal cord compression, Parkinsonism.

Clinical evaluation revealed impairment in activities of cognitive/intellectual development and behavioral/psychiatric disorders in all subjects with data available (n = 9). Language/speech impairment (dysarthria) was found in eight patients (88%), fine motor and gross motor impairments were reported in seven (77%) and five (55%) patients, respectively. Cognitive delay and impairment activities of daily life were seen in seven individuals (77%). Other signs related to behavioral disorders included learning disability, autism spectrum‐like behavior, depression, and aggressive behavior (two individuals). Anxiety was reported in three patients, and obsessive compulsive disorder was documented in only one case.

A summary of all aspects evaluated is presented in Tables 2 and 3.

## 4 DISCUSSION AND CONCLUSION

GM1 gangliosidosis is a rare and heterogeneous disease with a few studies focused on the adult form (type III). Here we report clinical findings in 10 adults GM1 gangliosidosis patients from Brazil.

Although no prospective natural history studies have been published for GM1 gangliosidosis thus far, several case series of juvenile and adult GM1 gangliosidosis describe the inexorable and irreversible neurological deterioration that progresses until death, which occurs at variable ages in the adult form.^7^, ^11^, ^18^, ^20^, ^23^, ^24^

In 2004, Muthane et al described three patients from India and revised 40 other previously reported subjects, reinforcing that the most frequent clinical findings were generalized dystonia with prominent facial dystonia and severe speech disturbances. Roze et al^24^ described four new patients and analyzed data from 44 other meticulously selected subjects from 16 Japanese and 15 non‐Japanese families. Clinical manifestations occurred before 20 years of age in most patients, typically presenting with gait disorders and/or speech disturbances, showing wide variations in severity and progression rates. Brunetti‐Pierri and Scaglia^6^ revised clinical, molecular, and management aspects in 209 subjects with all types of GM1 gangliosidosis, comprising 130 infantile, 23 juvenile, and 56 adult patients. Signs and symptoms of CNS involvement were invariably present in all cases, with a predominance of hypotonia and development delay in the infantile form in contrast with extrapyramidal, gait disturbances, speech difficulties, and dystonia in the adult form. Long‐term survival in the late‐onset phenotype varies greatly.^7^, ^14^ In 2015, Kannebley et al described 12 subjects from 10 unrelated Brazilian families from the region of Campinas (state of Sao Paulo) and from the Minas Gerais state, who presented with juvenile (n = 4) and adult (n = 8) GM1 gangliosidosis. The authors found that the clinical presentation is highly variable among individuals, and skeletal deformities and neurologic symptoms occurred at similar frequencies as initial features, but a combination of both was seen in all individuals over time.

The *GLB1* mutations found in our patients showed extensive molecular heterogeneity, posing a challenge to the study of genotype‐phenotype correlations. In the Brazilian population, Sperb et al^22^ reviewed 32 patients with all types of GM1 gangliosidosis from different regions of Brazil who were diagnosed at their reference laboratory and included clinical and molecular analyses. The series included five subjects with the infantile, 15 with the juvenile, and nine with the adult form. Once again, a genotype‐phenotype correlation could not be established for most patients.

According to the literature, the most frequent mutations are p.Arg59His and c.1577dupG, which, in homozygosis, have been associated with the early infantile phenotype.^21^ The p.Arg59His mutation was present in two of our patients. This mutation was first described by Morrone et al^19^ in an Italian patient. Santamaria et al^20^ and Sperb et al^22^ observed this allele in 27.9% and 19% of patients, respectively. The other frequent mutation (c.1577dupG) was present in only one patient in our study. This mutation has only been reported in Brazilian patients so far, except for one patient from Uruguay described by Santamaria et al.^20^ This mutation is associated with cognitive delay and hypertonia in homozygous patients and ophthalmic findings in compound heterozygous patients.^18^, ^22^

Regarding the clinical features, the majority of patients (5/6) showed short stature and below normal weight. Signs of dysostosis multiplex were detected in all subjects who underwent a complete radiological evaluation, which is consistent with the literature.^6^, ^18^, ^23^, ^24^

Scoliosis was evident in 7/10 subjects. Roze et al^24^ described a similar proportion for this finding, with 65% in one series of Japanese patients and 82% in non‐Japanese patients. Kyphosis was diagnosed in 4/10 patients in our cohort, and our finding is consistent with results reported in the literature, where 4/9 patients with adult GM1 presented this phenotype.^18^

Hip dystonia and platyspondyly were present in only 4/9 patients in our cohort, contrasting with the report by Kannebley et al,^18^ who showed that 10/11 and 10/10 patients had hip dysplasia and platyspondyly, respectively (note: as informed in Tables 1 and 2 patients of the present report were included in the sample studied by Reference 18).

Among other signs typically described in lysosomal storage diseases, corneal clouding and hearing impairment were seen in only one patient in our cohort. According to Muthane et al,^23^ the corneal opacities, macula spots, and cataracts frequently observed in both infantile or late infantile/juvenile GM1 gangliosidosis are unusual in the adult form.

The majority of patients needed help to walk, mainly due to skeletal and neurological abnormalities. This finding has not been reported in any case series studies of adult GM1‐gangliosidosis. However, Deodato et al,^25^ described the results of miglustat treatment on three Italian patients with juvenile/adult GM1 gangliosidosis and showed that in the adult patient the treatment led to a progressive improvement in walking ability with an increase in the distance covered in the 6‐minute walk test.^25^

Neurological findings revealed dystonia (60%) and swallowing problems (60%) as being the most frequent abnormalities. This proportion is lower than in Japanese patients, where the adult patients had dystonia and swallowing problems in 85% and 96% of the cases, respectively.^24^ However, our result is higher than reported by Brunetti‐Pierri and Scaglia,^6^ who observed dystonia in 22% of their adult patients.

Dysarthria was diagnosed in most patients (88%) in our cohort and is a frequent symptom, occurring in over 90% of individuals with the adult form.^24^ This findings has been reported previously by Roze et al^24^ to occur in 96% in one cohort of Japanese and 100% of non‐Japanese patients.

Most individuals in this cohort had impaired daily living activities, mainly due to the gross and fine motor difficulties. Seven patients presented cognitive delay and five patients presented difficulties in self‐supporting tasks including eating and toileting. The frequencies of behavioral disorders found in this study were low. According to Kannebley et al,^18^ cognitive and behavioral deficits have rarely been reported in adult GM1, but it is possible that the severe motor impairment and frequent finding of anarthria precluded a thorough cognitive evaluation in this and previous cohorts.

Parkinsonism has been reported in case series varying from 7.5%^23^ to 48%^24^ of the patients but was not seen in our series. Kannebley et al^18^ also reported that no patient presented Parkinsonism.

We organized our findings based on patient age (as seen in Tables 2 and 3). In so doing, we expecting to identify early presenting and late‐presenting abnormalities. This would be helpful for the evaluation of therapies currently under development. Interestingly, other than aspects such as development of kyphosis or wheelchair need, we were not able to find a clear difference between younger and older patients, which suggests significant phenotypic variability and heterogeneity, even among patients with the same type of GM1 gangliosidosis. This broad phenotypic spectrum poses a challenge for the study of this inherited metabolic disease. We speculate that the evaluation of biomarkers could be more informative and should be pursued in future studies.

This study presents additional data about clinical findings in the adult form of GM1 gangliosidosis. The aim was to improve the understanding of type III GM1 gangliosidosis, as prospective studies of this adult form are lacking and difficult to perform. The rarity of gangliosidoses, with patients scattered across different centers, makes the study of larger samples challenging. However, larger samples will be important to better understand the natural course of the disease and evaluate the efficacy of therapies.

## ETHICS APPROVAL AND CONSENT TO PARTICIPATE

All authors confirm that the study include a statement on ethics approval and consent. All procedures followed were in accordance with the ethical standards of the responsible committee on human experimentation (institutional and national) and with the Helsinki Declaration of 1975, as revised in 2000. The study was approved by the Ethics in Research Committee of Hospital de Clínicas de Porto Alegre (HCPA), under the reference number 15‐0281. All the patients and/or their guardians signed the Informed Consent Form (ICF).

## CONFLICT OF INTEREST

Luciana Giugliani, Carlos Eduardo Steiner, Chong Ae Kim, Charles Marques Lourenço, Mara Lucia Schmitz Ferreira Santos, Carolina Fischinger Moura de Souza, Ana Carolina Brusius‐Facchin, Guilherme Baldo, Mariluce Riege, Roberto Giugliani declare that they have no conflict of interest.

## ANIMAL RIGHTS

This article does not contain any studies with animal subjects performed by the any of the authors.

## AUTHOR CONTRIBUTIONS

L.G.: Participation in the study design, contributed to the concept and planning of the project, collection of data, study logistics, data analysis, creation of the first draft; G.B.: Participation in the study design, supervision of all stages, revision and correction of the first draft, review of the final version; R.G. (guarantor): Participation in the study design, supervision of all stages, revision and correction of the first draft, review of the final version; A.C.B.‐F.: Participation in the study design, review the molecular testing and their nomenclatures, review of the final version; M.R.: Participation in the study design, supervision of all stages, revision and correction of the first draft, review of the final version; C.E.S.: Participation in the study design, collection of data, review of the final version; C.A.K.: Participation in the study design, collection of data, review of the final version; C.M.L.: Participation in the study design, collection of data, review of the final version; M.L.S.F.S.: Participation in the study design, collection of data, review of the final version; C.F.M.S.: Participation in the study design, collection of data, review of the final version; All authors had a substantial contributions to conception and design, or acquisition of data, or analysis and interpretation of data; drafting the article or revising it critically for important intellectual content; final approval of the version to be published; and agreement to act as guarantor of the work (ensuring that questions related to any part of the work are appropriately investigated and resolved).
